# Pure stress urinary incontinence: analysis of prevalence, estimation of costs, and financial impact

**DOI:** 10.1186/s12894-019-0468-2

**Published:** 2019-06-04

**Authors:** Emanuele Rubilotta, Matteo Balzarro, Antonio D’Amico, Maria Angela Cerruto, Silvia Bassi, Chiara Bovo, Valerio Iacovelli, Daniele Bianchi, Walter Artibani, Enrico Finazzi Agrò

**Affiliations:** 1 Department of Urology, Azienda Ospedaliera Universitaria Integrata (AOUI), Piazzale Aristide Stefani, 1, 37126 Verona, Italy; 2 Healthcare Department Administrator, Azienda Ospedaliera Universitaria Integrata (AOUI), Verona, Italy; 3 Department of Experimental Medicine and Surgery, Urology Unit, Tor Vergata University, Rome, Italy

## Abstract

**Background:**

The prevalence of pure stress urinary incontinence (P-SUI) and the role of urodynamic investigation (UDI) prior to surgery for stress urinary incontinence (SUI) is debated. Since the exact prevalence of P-SUI is not clear, its clinical and economic impact is not well defined. The aims of this study were to evaluate the prevalence of P-SUI in a population of women who underwent UDI for urinary incontinence (UI), also assessing: 1) the correspondence between clinical diagnosis of P-SUI and urodynamic findings; 2) the analysis of costs in terms of UDI and eventually post-UDI avoided surgical procedures.

**Methods:**

A single cohort of women who underwent UDI for UI between January 2012 and July 2016 was prospectively collected and retrospectively analyzed. Clinical P-SUI was defined by the strict criteria of the International Continence Society. For each patient, history, physical examination and UDI were collected. The correspondence between clinical and urodynamic findings of P-SUI was analyzed. The rate of clinical P-SUI changed after performing UDI and the number of unnecessary intervention after UDI were reported. A wide cost analysis of UDIs, and the amount of surgical procedures that were believed unnecessary after UDI was reported.

**Results:**

Stress urinary incontinence was present in 323/544 (59.4%) patients. The prevalence of clinical P-SUI was 20.7% (67/323), while the prevalence of complicated SUI (C-SUI) was 79.3% (256/323). After UDI, diagnosis of P-SUI decreased to 18.3% (59/232). In 10.2% of cases (6/59) the scheduled middle urethral sling (MUS) was suppressed after the UDI results because 3/6 cases had detrusor overactivity and urge incontinence, in 2/6 cases SUI was treated with a conservative management, in 1/6 case an important voiding dysfunction was detected. Considering the national reimbursement in our country, the cost of each UDI was 296.5 euros and the total amount was 17,493.5 euros. So far the surgery-related savings covered 61.7–105.0% of the costs of total number of UDIs performed in the uncomplicated patients.

**Conclusions:**

The prevalence of clinical P-SUI is relevant, involving about 20% of women with clinical SUI. Although the correspondence between clinical and urodynamic diagnosis was high, we demonstrated that UDI may help in some cases to avoid an inappropriate surgical treatment. Therefore, UDI prior to SUI surgery should be considered to achieve a correct diagnosis and a proper therapeutic strategy.

## Background

Uncomplicated stress urinary incontinence (SUI), also called pure stress urinary incontinence (PSUI), and the role of urodynamic investigation (UDI) prior to surgery for SUI have been under debate in the last years [1–3].

Since the exact prevalence of P-SUI is not clear, its clinical and economic impact is even not well defined. It has been calculated that in women with uncomplicated SUI and a confirmatory preoperative basic office evaluation, from 13 to 33 millions of US dollars could be saved annually by not performing UDI test [4]. Nevertheless, other authors reported that savings obtained by not performing UDI in these patients seem to be moderate [5].

Considering the cost of a single UDI, it has been debated if it is useful in women with uncomplicated SUI prior to surgical correction. Few papers showed that an office evaluation is a sufficient work-up for women with uncomplicated SUI, since their incontinence surgery outcomes were not inferior if compared to women who underwent UDI [6, 7]. However, the correlation between real prevalence of uncomplicated SUI and costs of clinical evaluation, UDI and surgical treatment has never been systematically analysed.

The aim of our study was to evaluate the prevalence of clinical P-SUI in a population of women who underwent UDI for urinary incontinence (UI), assessing the correspondence between clinical diagnosis of P-SUI and urodynamic stress incontinence. Our secondary end-point was to analyze the costs of UDI in women with P-SUI, and the number of surgical procedures that were believed unnecessary after UDI, including related money savings.

## Methods

A single cohort of women who underwent UDI for UI between January 2012 and July 2016 was prospectively collected and retrospectively analyzed. Data were obtained from an electronic database. Urodynamic tests were performed according to the Good Urodynamic Practice [7]. P-SUI was defined by the International Continence Society (ICS) criteria. All women with clinical P-SUI had no history of: (i) previous pelvic surgical treatments; (ii) pain; (iii) aematuria; (iiii) recurrent infections; (iiiii) voiding symptoms; (iiiiii) pelvic irradiation; (iiiiiii) suspected fistula. Inclusion criteria were: naive patients for SUI without pelvic organ prolapse.

All women underwent UDI and their data were assessed by a urologist skilled in UDI and female urology. For all the patients, the first evaluation was obtained in the outpatient clinic recording patient’s history and physical exam. Though the history, the clinician assessed the prevalent type of incontinence and its duration, moreover urinalysis and urine culture were analyzed. The physical exam included abdominal, pelvic and vaginal examination, a stress test (with an ultrasound estimated bladder volume of 200 ml, and if negative with a bladder volume of 300 ml) and uroflowmetry with post-voiding residual volume [8]. Then, in case of sterile urine culture, a UDI was performed. Finally, the correspondence between clinical and urodynamic stress incontinence was evaluated.

UDI findings were compared with clinical data and the results were considered different when a UDI outcome was not correspondent to the pre-UDI expected clinical data. Clinical P-SUI was not confirmed when at least one of the following UDI observations was found: i) mixed urinary incontinence with predominant detrusor overactivity; ii) detrusor underactivity; iii) voiding dysfunction. Patients were considered to have a detrusor underactivity when Qmax was ≤12 ml/sec and Pdet/Qmax was ≤10 cmH2O [9]. Women were classified as having voiding dysfunction when Qmax was ≤12 ml/sec and Pdet/Qmax was ≥25 cmH2O [10, 11]. Depending on UDI results, surgical treatment options were discussed. Patients who had a change in the treatment strategy due to UDI results were recorded.

The exact cost of a single UDI was calculated with the support of the Clinical Management Department. The cost analysis included the human resources (one medical doctor and one nurse for each UDI) and all the materials used during the test. An additional economical evaluation was performed considering the National Health System reimbursement. Moreover, we calculated the total amount of expenses saved by not performing the scheduled surgical procedures of middle urethral sling (MUS) positioning, because they were believed unnecessary according to UDI results.

## Results

Clinical SUI was diagnosed in 323/544 (59.4%) patients. Prevalence of clinical P-SUI was 20.7% (67/323), while prevalence of complicated SUI (C-SUI) was 79.3% (256/323).

After UDI, diagnosis of P-SUI decreased to 18.3% (59/323). In 11.9% (8/67) of patients diagnosis of clinical P-SUI was not confirmed due to the detection of predominant detrusor overactivity (3 cases), detrusor underactivity (4 cases), and voiding dysfunction (1 case).

**Fig. 1:**
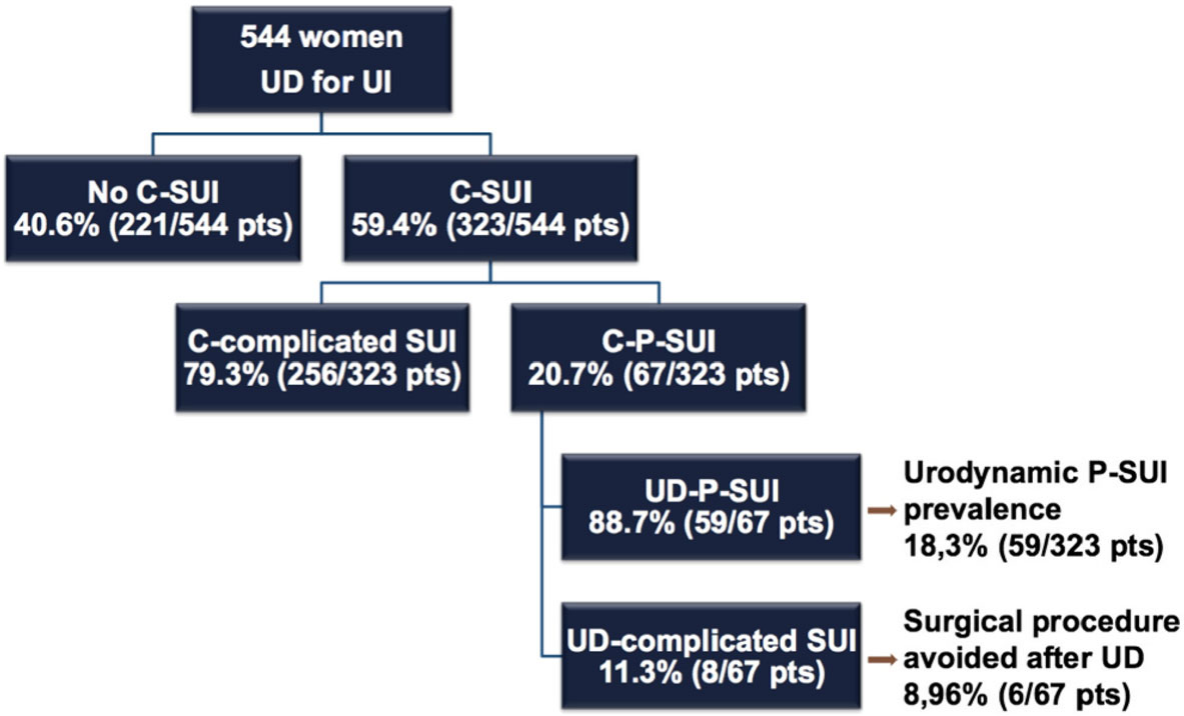
Patients stratification based on clinical and urodynamic diagnoses, including their impact on surgical indications. (Legend: UDI: urodynamic investigation; UI, urinary incontinence; C-SUI: clinical stress urinary incontinence; P-SUI: pure stress urinary incontinence)

In 8.96% of cases (6/67) the scheduled MUS was cancelled because of UDI results. In 3/6 patients detrusor overactivity and urge incontinence were detected, in 2/6 patients SUI was treated by pelvic floor rehabilitation, in 1/6 case an important voiding dysfunction was detected. Results are better shown in Fig. 1, which reports patients characteristics based on clinical and UDI diagnoses and details about surgical UI correction cancelled after UDI.

Being the Italian National Health System reimbursement for each UDI 296.5 €, the total cost for the 59 uncomplicated patients in our series was 17,493.5 €. If we consider a reimbursement by our National Health System ranging 1800–3073 € for each surgical procedure for female SUI (depending from the setting – Day Surgery or hospitalization for two or more days), the total amount saved thanks to UDI findings ranged 10,800–18,438 €. These savings cover 61.7–105.0% of the costs for total number of UDIs performed in the uncomplicated patients in our series.

## Discussion

In the literature few data are available on P-SUI with a prevalence ranging from 5.2 to 36% [1, 2, 12, 13] (Table 1). In our series, prevalence was about 20%, aligned with the results reported by Jeong [13].

**Table 1:** Pure stress urinary incontinence (P-SUI) prevalence, correspondence between clinical PSUI and urodynamic findings, and avoided surgery after urodynamic testing

	P-SUI prevalence	Correspondence	Avoided surgery
W. Agur et al., 2008	5.2%	–	–
S.J. Jeong et al., 2012 [13]	20.7%	79.1%	–
C.W. Nager et al., 2012 [1]	34%	43.2%	5.4%
M. Serati et al., 2015	36%	37.8%	9.5%
E. Rubilotta et al., 2019	20.7%	81.7%	8.96%

Some authors believe that the high prevalence of P-SUI could be secondary to restricted clinical skills [3]. For this reason, our lower rate might be related to a higher expertise in the field of urodynamics and female urology. Furthermore, this *know-how* could also explain the high correspondence between UDI findings and clinical data which is a relevant controversial topic in the literature (Table 1).

Some patients experienced a change in their therapeutic strategy after UDI, leading to a significant reduction of unnecessary surgical procedures (8.96%). Reversely, many women would have undergone an inappropriate surgical treatment (MUS) in case they did not perform UDI prior to surgery. Our results are aligned to those of Serati et al. who modified or cancelled the planned SUI surgery in 9.7 and 9.5% of cases, respectively [2].

In 2010, 260,000 MUS were implanted in the USA. If we consider a prevalence of clinical P-SUI of 20.7% (according to our study), we can therefore estimate that around 52,000 women underwent a MUS for P-SUI surgical correction [14]. Among these patients, almost 4659 (8.96%) might have undergone an unnecessary surgery if the UDI had not been administered before surgical treatment.

Thus, the issue of unnecessary surgical procedures seems to represent a major clinical and economic topic. Re-intervention rates related to voiding dysfunction or to mesh extrusion have been reported in 3.7% of cases [15]. Although with the limitations of this analysis, if we match such data with our results, an estimated number of 172 women may have experienced these complications due to an unnecessary surgical treatment. Moreover, considering the reported chronic pain rate of 4.1% after MUS, the estimated number of patients with this potential complication after an unnecessary surgery would be around 191. Considering literature data, 740 mesh removal procedures have been reported in peer-reviewed publications and 7654 meshes were removed in patients presenting with sling complications, thus leading to a total of 8394 complicated women after MUS [15]. Just in order to extend the analysis, if we apply our criteria to the population of the cited study [14], we confirmed the previously reported results: 1738 patients were supposed to have P-SUI and in 156 of cases the SUI surgical intervention could have been cancelled after UDI. These results would be even higher if we refer to the greater P-SUI prevalence reported by the other authors [1, 2].

In the light of these considerations, we believe that UDI may help in the clinical diagnostic process of all women presenting with SUI and not only in the complicated ones. Indeed, UDI might help to rule out unnecessary surgical treatments and their potential sequelae.

Cost analysis must contemplate both direct and indirect costs. The direct costs comprehend the total amount for a single UDI, for the surgical procedure, and for hospitalization. The negative externalities are those costs that occur in the complicated patients, including repeated UDI, use of devices, and potentially a new surgical treatment and hospitalization. Our estimated rate of unnecessary surgical treatment was 8.96%, meaning that without UDI these women would have had their insurance/health care to pay for their surgery without a confirmed indication. Nevertheless, insurance/health care would have paid even for the costs of the management of negative externalities.

In our study, we performed an analysis of the direct costs in a cohort of women with P-SUI. Analyzing the savings from the total number of UDIs performed in the uncomplicated patients in our series we found that costs of avoided surgical treatment covered the amount of money necessary for the UDIs. Regarding negative externalities, a complete cost evaluation should also include the potential legal actions due to complications after unnecessary interventions.

A limitation of our study may be related to the retrospective design, however all the data required were available for the analysis. A second limitation may be related to the different evaluations of the costs in diverse geographical areas.

## Conclusions

The prevalence of clinical P-SUI is relevant, involving 1/5 of women with clinical SUI. The correspondence between clinical and urodynamic diagnosis is high. Nevertheless, in some patients an inappropriate treatment was avoided basing on urodynamic results. Although UDI involves an economic burden, its role should be emphasized. Indeed, according to our data, UDI prior to SUI surgery is useful to avoid unnecessary surgical procedures and this finding should be taken into account when calculating the real costs/savings about performing or not UDI.
